# Willingness of patients with chronic diseases to use telepharmacy services in Bandung City, West Java, Indonesia

**DOI:** 10.1038/s41598-025-09688-3

**Published:** 2025-07-11

**Authors:** Sofa D. Alfian, Qisty A. Khoiry, Meliana Griselda, Ivan S. Pradipta, Nursiswati Nursiswati, Rizky Abdulah

**Affiliations:** 1 Department of Pharmacology and Clinical Pharmacy, Faculty of Pharmacy, Universitas Padjadjaran, Jl. Raya Jatinangor, KM 21, Jatinangor, Sumedang, Indonesia; 2 Drug Utilization and Pharmacoepidemiology Research Group, Center of Excellence for Pharmaceutical Care Innovation, Universitas Padjadjaran, Jatinangor, Indonesia; 3 Center for Health Technology Assessment, Universitas Padjadjaran, Jatinangor, Indonesia; 4 Faculty of Nursing, Universitas Padjadjaran, Jatinangor, Indonesia

**Keywords:** Telemedicine, Telepharmacy, Willingness, Practices, Indonesia, Chronic diseases, Risk factors, Health care

## Abstract

The COVID-19 is known to be a severe pandemic that has accelerated the adoption of telepharmacy services. However, little is known about the current practices and willingness of patients with chronic diseases in the new-norm, specifically in a setting where such services are not properly established before the pandemic. The main objective of this study was to assess the current practices and willingness to use telepharmacy services as well as to identify factors associated with low willingness among patients with chronic diseases in Bandung City in Indonesia. A multisite cross-sectional survey was conducted in seven community health centers (CHCs) and one hospital in Bandung City, in Indonesia, which were selected based on the highest number of patients with chronic diseases. The survey utilized a purposive sampling method among patients with hypertension, dyslipidemia, diabetes, chronic obstructive pulmonary diseases (COPD), and/or chronic kidney diseases. Current practices and willingness to use telepharmacy services were evaluated through a paper-based questionnaire. Binary logistic regression analyses were performed to determine factors associated with low willingness to use telepharmacy services. Odds ratios (ORs) with 95% confidence intervals (CIs) were reported. A total of 443 patients with chronic diseases were participated, with most having hypertension. Overall, both current practices and willingness to use telepharmacy services were low. Factors associated with low willingness included being 50 years or older (OR = 3.41; 95%CI = 1.46–7.97), inability to use the internet (OR = 3.92; 95% CI = 1.12–13.76), using smartphones independently (OR = 3.64; 95% CI = 1.13–11.72) or with assistance (OR = 6.75; 95% CI = 1.47–31.07), and daily phone usage of less than an hour (OR = 5.50; 95% CI = 1.08–27.89). The study indicated low practices and willingness to use telepharmcy services among patients with chronic diseases. As a result, patients-specific factors such as older age, inability to use the internet, as well as low daily use of smartphones that associated with the low willingness to use telepharmacy services should be considered in order to facilitate telepharmacy implementation in Indonesia.

**Supplementary Information:**

The online version contains supplementary material available at 10.1038/s41598-025-09688-3.

## Introduction

The COVID-19 pandemic has changed the way pharmacists provide pharmaceutical care to patients^1^. Telepharmacy services, which is known to be the use of digital technology to offer pharmaceutical care remotely, have been proposed as an adaptive method to ensure the continuation of health care services during the adoption of several restrictions^2,3^. The technological method offers a wide range of services, including drug prescribing and dispensing, pharmacists counseling, patients monitoring, education, and clinical care^4^. Telepharmacy is defined as the provision of pharmaceutical care services through the use of telecommunications and digital technologies^5^. This encompasses a broad range of remote services, including but not limited to, pharmacist consultations (e.g., medication counseling, patient education), electronic prescribing, online medication ordering and dispensing, and virtual follow-up care aimed at optimizing medication use and patient outcomes^6^.

Prior to the COVID-19 pandemic, several developed countries were already familiar with telepharmacy, which predominantly focused on improving healthcare access in rural areas^7^. For instance, as of 2018, 76% of hospital systems in the United States had implemented some form of telepharmacy^8^. Norway has emerged as a leader in this field, boasting over 30 years of experience and instituting lasting regulatory changes to support telepharmacy implementation^9^. Factors influencing this uptake include the technological capabilities of electronic medical records and supportive reimbursement policies^10^. While telepharmacy remains a relatively novel concept in Indonesia, the Indonesian Ministry of Health (MoH) has begun to integrate telemedicine and telepharmacy into the healthcare system. The Ministry released Regulation Number 20 of 2019, which pertains to the implementation of telemedicine and telepharmacy services between healthcare facilities^11^. However, the scope of this regulation is limited to inter-facility interactions.

However, the pandemic necessitated a rapid shift in the utilization of telepharmacy to address an immediate crisis—mitigating the transmission of COVID-19 globally, including in Low- and Middle-Income Countries (LMICs)^7^. Launched in April 2020, telepharmacy services in India enabled 3 million consultations by March 2021, with over 20% of patients using the app multiple times, highlighting its potential to maintain continuity of care^12^. In the Southeast Asian region, some countries, such as Thailand and Vietnam, had also begun exploring and implementing telepharmacy initiatives, often driven by similar aims of expanding access and improving healthcare efficiency^13^. Telepharmacy in Thailand is currently undergoing development and standardization, establishing foundational guidelines for services and pharmaceutical care, alongside an information architecture to support operations, as the existing standards from the Pharmacy Council require further detailed implementation^14^. In Vietnam, despite being a new concept without relevant legal regulations, telepharmacy is anticipated to develop in alignment with healthcare demands and the evolution of electronic health systems, with Ho Chi Minh City playing a pivotal role in leading the implementation of information technology in the healthcare sector through its “Smart Health” project for the period of 2021 to 2025, which aims to enhance services and elevate public satisfaction via innovation^15^. Notably, as of today, nearly 87% of surveyed pharmacists in Vietnam are utilizing telepharmacy in their practice, primarily through phone calls and Zalo, a Vietnamese social media platform with 100 million users^15^. In Indonesia, various private telepharmacy providers supported the MoH regulations during the pandemic by offering their telemedicine platforms for both COVID-19 public health responses and routine healthcare services^16^. In particular, the Indonesian MoH issue a decree (No HK.01.07/MENKES/ 4829/2021) that enabled healthcare services via telemedicine^17^ and the Indonesian Medical Council issued Regulation No. 74 of 2020 concerning the Clinical Authority and Medical Practice through Telemedicine during the COVID-19 Pandemic^18^. While these regulations were temporary and expired upon the official termination of the COVID-19 public health emergency by the Indonesian government^19^, they played a crucial role in establishing initial operational models and demonstrating the potential of telepharmacy, thereby providing a foundation for future regulatory and service development. This foundation has been built upon by subsequent regulations.

In the post-pandemic era, the Indonesian MoH has taken further steps to ensure the safety of telemedicine services by establishing a new regulatory decree: the Decree of the Minister of Health (KMK) Number HK.01.07/MENKES/1280/2023. This decree aims to develop a digital health innovation ecosystem through a regulatory sandbox^20^, designed to protect both the public and telehealth industry by mitigating risks associated with telemedicine, such as data security and patient safety. The Indonesian Food and Drug Authority Regulation No. 8 of 2020 specifically addresses the online distribution of medicines, stipulating that pharmacies must work with licensed e-pharmacy platforms^21^. More significantly, the recently issued Government Regulation No. 28 of 2024 concerning the Implementation of the Health Law officially incorporates telepharmacy as a defined component within telemedicine services^22^. The COVID-19 outbreak was a game changer, creating a new norm that became an integral part of this transformative period, significantly impacting pharmacy practices. Even now, telepharmacy continues to address ongoing barriers to primary care in the Southeast Asia region^23^. In Indonesia, telepharmacy services are currently accessible through various platforms provided by both public and private sectors, often supported by partnerships with local healthcare providers^24^. These platforms provide a range of services, including consultations with pharmacists, locating nearby pharmacies, purchasing medications online, and accessing educational materials (such as webinars and e-modules)^25,26^. So far, telepharmacy implementation in Indonesia includes several licensed e-pharmacy platforms that work with licensed pharmacies, enabling online consultations with pharmacists, e-prescribing, and the online purchase of over-the-counter medications. Examples include GoApotik, K24Klik, and Kimia Farma Mobile. Regarding patient accessibility, GoApotik functions like a marketplace specifically for pharmacies, with 7000 pharmacy outlets and reaching 480 cities in Indonesia^27^. Meanwhile, K24Klik is the telepharmacy platform of “Apotek K24” (brand name of pharmacy outlet), which is the largest national private pharmacy chain in Indonesia, operating 24 h non-stop with 750 outlets spread across 156 cities and 29 provinces in Indonesia^28^. Kimia Farma Mobile is the telepharmacy platform of "Apotek Kimia Farma" (brand name of pharmacy outlet), which is part of the State-Owned Enterprise Pharmaceutical Holding in Indonesia, with 1,234 pharmacy outlets spread across 37 provinces in Indonesia^29^.

Telepharmacy has been shown to help both patients and healthcare providers by lowering diseases exposure, enhancing healthcare accessibility, making better use of resources, and producing beneficial health and behavioral results^30^. These benefits showed higher impacts in patients with chronic diseases due to the necessity for long-term medication use^31,32^. For instance, telepharmacy promotes quality treatment, increases healthcare provider and patients engagement^33^, as well as improves self-management^34^, and reduces difficulties in medication refills^10^. Notably, 35.4% of patients cited reduced waiting times as the primary reason for their preference for hospital telepharmacy services^35^. In Indonesia in 2024, over 2413 hospitals and 2692 Community Health Centres offer telepharmacy services^36^. A survey of 784 patients in Indonesia who used telemedicine during the pandemic revealed that 81% intended to continue utilizing telepharmacy services even after the pandemic ended^37^. This significant transition may not be transitory, needing the engagement of both pharmacists and patients in telepharmacy services.

Despite its benefits, telepharmacy services remain underutilized^25,38^, particularly in developing countries that lacked well-established telepharmacy infrastructure before COVID-19. For instance, in Bahrain, just half of chronic diseases patients (142; 57%) experienced willingness to use such services in the future^39^. Previous investigations have found that chronic diseases treatment by telepharmacy and in-person therapy yields comparable clinical results^40^, but the lack of in-person options may discourage the usage of telehealth^41^. This challenge needs to be addressed, particularly in settings with a high prevalence of chronic diseases such as Indonesia^42^. Understanding the current practices and willingness to use telepharmacy services among patients with chronic diseases as potential users in the long term is important, particularly because these patients require continuous, often lifelong, medication management and monitoring, a unique need that telepharmacy could significantly impact. However, previous studies in Indonesia solely focused on assessing willingness to use telepharmacy services among pharmacists^43,44^, the general population^45^, or pharmacy students^46^, there remains a gap in understanding the specific drivers of telepharmacy adoption among chronic disease patients in Indonesia. Our study directly addresses this gap by exploring their current practices and willingness to use these services, thereby providing insights to develop effective and patient-centered strategies for the adoption of such services. Therefore, this study aims to assess the current practices and willingness to use telepharmacy services as well as to identify factors associated with the willingness among patients with chronic diseases in Indonesia.

## Methods

This study was reported in accordance with the guidelines of Strengthening the Reporting of Observational Studies in Epidemiology (STROBE) for cross-sectional survey^47^ (Table S1, Supplementary data).

### Study design, setting, and study population

A multisite cross-sectional survey was carried out at seven community health centers (CHCs) and one hospital in Bandung City, in Indonesia, which were selected based on the highest number of patients with chronic diseases^48^. This analysis was conducted between December 2023 and February 2024. Patients were eligible when they were at the age of 18 years and above, had either experience using telepharmacy or no prior experience, and diagnosed with at least one chronic diseases such as diabetes, hypertension, dyslipidemia, stroke, hypercholesterolemia, chronic kidney diseases or kidney failure, cardiovascular diseases, gastrointestinal diseases, optical diseases, gout diseases, neurological diseases, asthma, tuberculosis, and/or chronic obstructive pulmonary diseases (COPD) that were identified through registered medical records. Furthermore, patients who had not lived in Indonesia for the past three years, as well as those with communication and hearing issues were excluded.

### Ethical approval

The study was approved by the Health Research Ethics Committee of Universitas Padjadjaran, Indonesia (No. 614/UN6.KEP/EC/2022). A written informed consent was obtained before proceeding with the survey. This study adhered to all relevant guidelines and regulations, including the Declaration of Helsinki.

### Data collection

Data was collected using a purposive sampling method for the selection of study sites and identification of potential participants. Seven CHCs and one hospital in Bandung City were purposively selected based on the highest number of patients with chronic diseases^48^, ensuring that the study accessed information-rich contexts highly representative of chronic disease patient management within healthcare system in Bandung City. Purposive sampling was specifically chosen over probabilistic methods to ensure the selection of healthcare centers with the highest number of chronic diseases patients, thereby maximizing access to our target population and providing rich and relevant data, which is critical for understanding their practices and willingness regarding telepharmacy services. Within these selected sites, specific groups of eligible chronic disease patients were targeted. At the seven CHCs, all registered participants of the Chronic Disease Management Program were approached to participate in the study. At the hospital, chronic disease patients waiting in the pharmacy queue were approached. Eligible patients from these targeted groups were informed about the study and invited to participate voluntarily by completing a paper-based questionnaire which was adapted from previous studies^49–51^.

The questionnaire was estimated to be completed in 15 min and was grouped into three parts, including (i) sociodemographic, health-related, and smartphones-related information, (ii) current practices of telepharmacy services, and (iii) willingness to use telepharmacy services. The sociodemographic information included age, gender, and health-related information, particularly the type(s) and duration (in years) of chronic diseases. Meanwhile, smartphones-related information included the ability to use the internet and smartphones or cell phone (unable to use independently, need assistance, or ability to use independently), duration of phones use per day (less than an hour, 1–4 h, or more than 5 h), and internet availability at home (yes or no). We made a distinction between smartphones and cellphones, as both devices continue to be widely used in Indonesia. A cellphone is a basic mobile device primarily designed for making and receiving voice calls and text messages. In contrast, a smartphone is a more advanced mobile device that provides a broad range of capabilities, including internet access, application usage, and multimedia functions, extending beyond the basic communication features of a cellphone.

Questions regarding current practices on telepharmacy services were based on a previous study^51^ that consisted of 12 questions about frequency and the type of information obtained from such services (Table S2, in Supplementary data). Patients who answered yes to at least seven questions were considered to have high practices of telepharmacy^51^. Similarly, questions on willingness to use telepharmacy services were based on a previous study^51^ which was measured by 16 questions including three questions with a score of 4 (0–3), one question with a score of 3 (1–3), and the remaining 12 were “yes or no” questions (yes = 1 and no = 0) (Table S3, in Supplementary data). Accordingly, the total score was 24 points, with scores ≥ 13 considered as having a high willingness to use telepharmacy services^51^.

This study adhered to the guidelines established by The International Society for Pharmacoeconomics and Outcome Research for questionnaire adaptation^52^ which included the initial translation of the questionnaire into Indonesian (forward translation) and subsequent re-translation into English (back translation), both performed by independent professional language translators. The translated version of the questionnaire was then shared with three clinical pharmacy experts for their evaluation. The opinions of these experts regarding the clarity of each item were considered before finalizing the questionnaire. To ensure content and face validity, a pilot study was conducted among 30 patients diagnosed with chronic diseases to assess the comprehensibility and clarity of the questionnaire. The validity of the Indonesian questionnaire was shown through the correlation values between each question and the total score which exceeded the threshold of 0.098 for practices and willingness to use telepharmacy services. Moreover, the reliability of the questionnaire was confirmed with Cronbach Alpha coefficients of 0.878 for practices and 0.899 for willingness to use telepharmacy services, respectively.

### Sample size calculation

The sample size was calculated using the formula specified for the cross-sectional survey^53^ with the assumption of 95% CIs of z = 1.96 and d = 0.05, with the proportion of chronic diseases at 71% among the Indonesian population^54^. A minimum sample size of 357 patients would be needed when there was an unusable response of 10%. The final sample size analyzed was 443, exceeding the calculated minimum.

### Data analysis

Characteristics of patients were summarized through the use of descriptive statistics. Bivariate analysis was conducted using chi-square to assess the association between independent variables and outcome. Variables with *p* < 0.05 were included in the initial model on multivariate analysis. Multivariate logistic regression analysis, which was performed to assess the association of patients’ characteristics with low willingness to use telepharmacy services, using backward elimination to obtain the odds ratio (OR) along with 95% CIs. A subgroup analysis was conducted to investigate willingness to use telepharmacy, comparing chronic disease patients who had never used telepharmacy and those who had prior experience. Patients were specifically classified as have never used telepharmacy if they provided a “No” response to all questions within the questionnaire related to telepharmacy practice. A *p*-value of < 0.05 was considered significant, and all statistical tests were performed using SPSS software (version 27.0; IBM, Armonk, NY, USA).

## Results

### Characteristics of patients

**Fig. 1:**
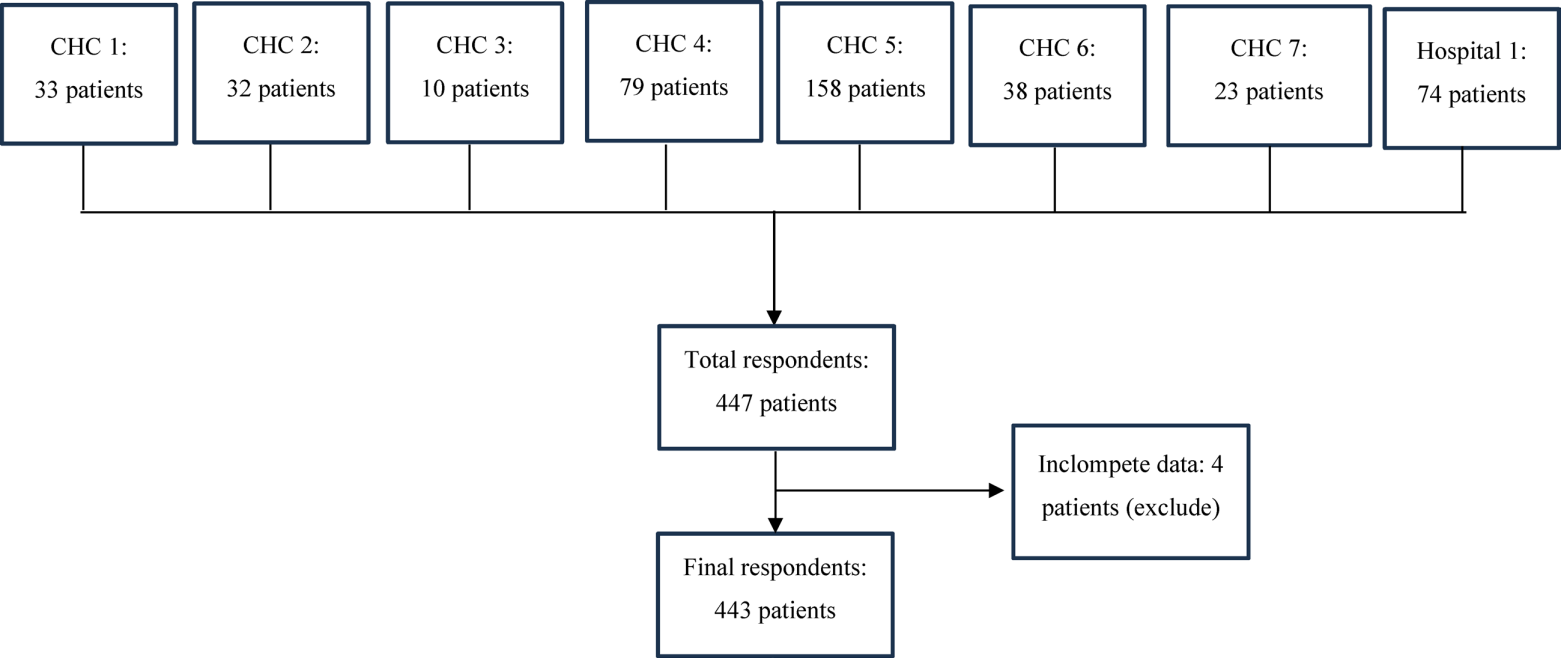
Flowchart of Patients Selection at Each Site.

A total of 443 patients were included in this study with a response rate of 100% (Fig. 1). The majority of patients were above 60 years old (306, 69.1%), male (347, 78.3%), and had been diagnosed with chronic diseases for five years or more (288, 65.0%). Most of them had the ability to use the internet (213, 48.1%), cell phones (273, 61.6%), and smartphones (260, 58.7%), with the duration of using smartphones for about 1 to 4 h per day (372, 84.0%). However, more than half of patients stated that they do not have internet access at home (264, 59.6%). The leading chronic diseases was hypertension (158, 35.6%), which was followed by diabetes (76, 17.1%) and the combination of these two ailments (54, 12.1%) (Table S4, Supplementary Data). A total of 258 patients (58.0%) had a single chronic disease and 185 patients (42.0%) had multiple chronic diseases (Table S4, Supplementary Data).

### Current practices of telepharmacy services

The overall level of practices of telepharmacy services among most patients (406, 91.6%) was reported to be low, with only a small percentage capable enough to use technology (37, 8.4%) (Table 1). Almost all of patients stated they did not have any experience in using telepharmacy services, as shown in Table 2. Furthermore, most of patients had never searched the internet for any information about diseases or related treatment (323, 72.9%), the doctor they were having appointments with (364, 82.2%), recommended lifestyle (327, 73.8%), and the types of chronic diseases being affected with (351, 79.2%). Most of the patients also reported that they had never renewed the prescription online (404, 91.2%), had never had an online consultation with a doctor (396, 89.4%), had never accessed the medical history online (421, 95.0%), and had never received any online medical check-up (419, 94.6%). In terms of using medical devices that connect to mobile or website applications, most of them reported no experience in using such devices (415, 93.7%). Moreover, patients stated they had never posted about the health conditions on any online platforms (407, 91.9%) and never joined any online social network related to their health conditions (329, 74.3%). Lastly, the majority of them had never made online appointments with any doctors (413, 93.2%).

**Table 1:** Characteristics of the patients (N = 443).

No	Characteristics	N (%)
1	Age (in years)	
	< 50	32 (7.2)
	51–60	105 (23.7)
	> 60	306 (69.1)
2	Gender	
	Male	347 (78.3)
	Female	96 (21.7)
3	Disease duration (in years)	
	< 5	155 (35.0)
	≥ 5	288 (65.0)
4	Use of internet	
	Yes, on my own	213 (48.1)
	Yes, with the help of others	87 (19.6)
	No	143 (32.3)
5	Use of cell phone	
	Yes, on my own	273 (61.6)
	Yes, with the help of others	56 (12.6)
	No	114 (25.7)
6	Use of smartphone	
	Yes, on my own	260 (58.7)
	Yes, with the help of others	55 (12.4)
	No	128 (28.9)
7	Duration of phone use in a day (in hours)	
	< 1	56 (12.6)
	1–4	372 (84.0)
	> 4	15 (3.4)
8	Availability of internet access at home	
	Yes	179 (40.4)
	No	264 (59.6)
9	Willingness	
	High	101 (22.8)
	Low	342 (77.2)
10	Practice	
	High	37 (8.4)
	Low	406 (91.6)

**Table 2:** Current practice of telepharmacy among patients with chronic diseases (N = 443).

No	Questions	N (%)
1	Have you ever searched online for information about a disease or medical problem that you have?	
	Yes	120 (27.1)
	No	323 (72.9)
2	Have you ever searched online for information about the doctor you are having appointments with?	
	Yes	79 (17.8)
	No	364 (82.2)
3	Have you ever typed information on an application about your dietary, physical exercise, and overall lifestyle modifications?	
	Yes	116 (26.2)
	No	327 (73.8)
4	Have you ever typed information on an application about a chronic illness you have?	
	Yes	92 (20.8)
	No	351 (79.2)
5	Have you ever renewed a prescription online?	
	Yes	39 (8.8)
	No	404 (91.2)
6	Have you ever consulted your doctor/pharmacist online?	
	Yes	47 (10.6)
	No	396 (89.4)
7	Have you ever used a personal online health record for your health?	
	Yes	22 (5.0)
	No	421 (95.0)
8	Have you ever looked at a medical test result online?	
	Yes	24 (5.4)
	No	419 (94.6)
9	Have you ever used a device that measures health information (like blood pressure; and blood glucose level) that connects to your mobile/website application?	
	Yes	28 (6.3)
	No	415 (93.7)
10	Have you ever posted anything online about your health condition or health care?	
	Yes	36 (8.1)
	No	407 (91.9)
11	Have you ever joined an online group that is for a health issue that you have?	
	Yes	114 (25.7)
	No	329 (74.3)
12	Have you ever booked an appointment with doctors/pharmacists online?	
	Yes	30 (6.8)
	No	413 (93.2)

### Willingness to use telepharmacy services

**Fig. 2:**
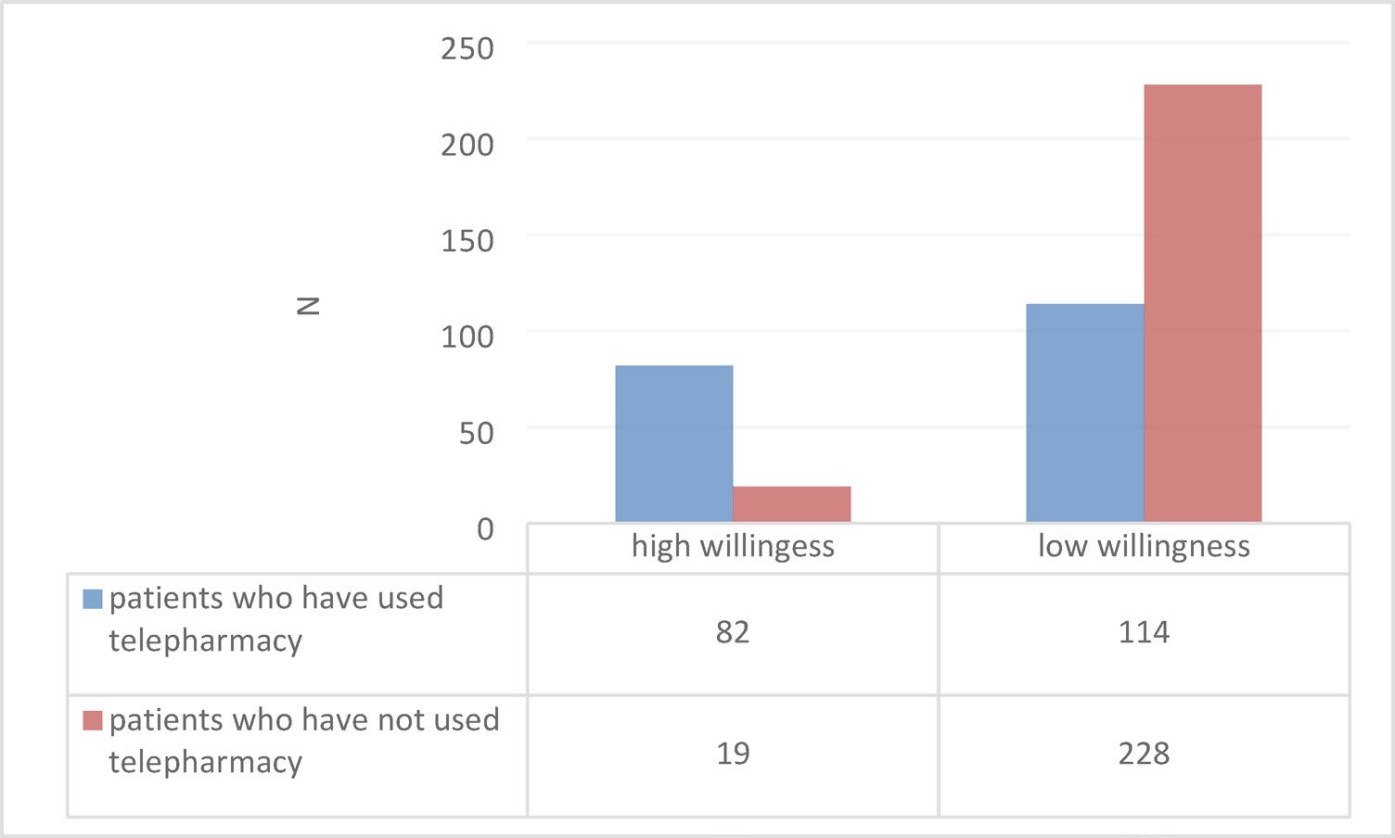
Distribution of Patient Willingness to Use Telepharmacy by Prior Telepharmacy Use.

The overall level of willingness to use telepharmacy services among most patients (342, 77.2%) appeared to be low (Table 1). Almost all patients (403, 91.0%) were confident about their health management, as presented in Table 3. In terms of telepharmacy, they possessed low awareness and interest (Table 3). Nearly all patients showed no familiarity with telepharmacy (363, 81.9%) and no interest in tracking information about chronic diseases (311, 70.2%), diet and calorie tracking (315, 71.1%), exercise tracking (314, 70.9%), prescription history tracking (319, 72.0%), and medical test reminders (314, 70.9%). They also showed low interest in using telepharmacy even when recommended by doctors (342, 77.2%), hospitals (342, 77.2%), pharmacists (351, 79.2%), health insurance (354, 79.9%), the government (358, 80.8%), nurses (354, 79.9%), or technological company (360, 81.3%). Moreover, most patients were very worried about the privacy and confidentiality of their personal information being recorded online (146, 33.0%) and were not interested in using application-or-website-based telepharmacy (228, 51.5%). Among those who have no prior experience with telepharmacy, an overwhelming majority (228 patients, approximately 92.3%) report low willingness, while very few (19 patients, approximately 7.7%) report high willingness. In contrast, among those who have used telepharmacy, more patients report low willingness (114 patients) than high willingness (82 patients), though a substantial number still have high willingness (approximately 41.8%) (Fig. 2).

**Table 3:** Willingness to use telepharmacy services (N = 443).

No	Statements and Questions	N (%)
1	Confident toward your own health management	
	Very confident	403 (90.9)
	Somewhat confident	30 (6.8)
	Not too confident	5 (1.1)
	Not at all confident	5 (1.1)
2	Familiarity with telepharmacy services	
	Yes	51 (11.5)
	Yes, I have heard, but I do not know the details	29 (6.6)
	No	363 (81.9)
3	Are you interested in tracking information about your chronic illness?	
	Yes	132 (29.8)
	No	311 (70.2)
4	Are you interested in tracking information about your diet and calories	
	Yes	128 (28.9)
	No	315 (71.1)
5	Are you interested in tracking information about your exercise	
	Yes	129 (29.1)
	No	314 (70.9)
6	Are you interested in getting reminders to take your prescriptions	
	Yes	124 (27.9)
	No	319 (72.0)
7	Are you interested in getting reminders to take health tests?	
	Yes	129 (29.1)
	No	314 (70.9)
8	Would you be interested in using this type of telepharmacy if it were from your doctor?	
	Yes	101 (22.8)
	No	342 (77.2)
9	Would you be interested in using this type of telepharmacy if it were from the hospital you use?	
	Yes	101 (22.8)
	No	342 (77.2)
10	Would you be interested in using this type of telepharmacy if it were from a pharmacist?	
	Yes	92 (20.8)
	No	351 (79.2)
11	Would you be interested in using this type of telepharmacy if it were from your health insurance plan?	
	Yes	89 (20.1)
	No	354 (79.9)
12	Would you be interested in using this type of telepharmacy if it were from a government group?	
	Yes	85 (19.2)
	No	358 (80.8)
13	Would you be interested in using this type of telepharmacy if it were from a nurse?	
	Yes	89 (20.1)
	No	354 (79.9)
14	Would you be interested in using this type of telepharmacy if it were from a company like Google or Apple?	
	Yes	83 (18.7)
	No	360 (81.3)
15	In general, if your health information were online, how worried would you be about the privacy and confidentiality of your information?	
	Very worried	146 (32.9)
	Somewhat worried	112 (25.3)
	Not too worried	54 (12.2)
	Not at all worried	131 (29.6)
16	Currently, how much you are interested in using telepharmacy (application, website)?	
	Very interested	107 (24.2)
	Interested	60 (13.5)
	A little bit interested	48 (10.8)
	Not at all interested	228 (51.5)

### Factors associated with low willingness to use telepharmacy services

Age, internet use, cell phone use, smartphone use, duration of daily phone use, and availability of internet access at home were initially identified as potentially associated with the low willingness to use telepharmacy based on the findings of Chi-square bivariate analyses (Table 4). Based on multivariate analysis, being aged 51 to 60 years old (OR = 3.41; 95% CI = 1.46–7.97) or older than 60 years (OR = 3.74; 95% CI = 1.68–3.35), the inability to use the internet (OR = 3.92; 95% CI = 1.12–13.76), ability to use smartphones independently (OR = 3.64; 95% CI = 1.13–11.72), ability to use smartphones with the help of others (OR = 6.75; 95% CI = 1.47–31.07), and daily phone usage less than an hour (OR = 5.50; 95% CI = 1.08–27.89) were associated with a lower willingness to use telepharmacy services (Table 4).

**Table 4:** Bivariate and multivariate analysis of factors associated with the low willingness to use telepharmacy (N = 443).

No	Characteristics	Bivariate			Multivariate	
		High willingness (N = 101)	Low willingness (N = 342)	*p*-value	Odds ratio (95% CI)	*p*-value
1	Age (in years)			< 0.001^a^		
	< 50	17 (16.8)	15 (4.4)		Ref	
	51–60	26 (25.7)	79 (23.1)		3.41 (1.46–7.97)	0.005^b^
	> 60	58 (57.4)	248 (72.5)		3.74 (1.68–3.35)	0.001^b^
2	Gender			0.975		
	Male	79 (78.2)	268 (78.4)			
	Female	22 (21.8)	74 (21.6)			
3	Disease duration (in years)			0.936		
	< 5	35 (34.7)	120 (35.1)			
	≥ 5	66 (65.4)	222 (64.9)			
4	Use of internet			0.004^a^		
	Yes, on my own	60 (59.4)	153 (44.7)		2.06 (0.94–4.50)	0.071
	Yes, with the help of others	22 (21.8)	65 (19.0)		Ref	
	No	19 (18.8)	124 (36.3)		3.92 (1.12–13.76)	0.033^b^
5	Use of cell phone			< 0.001^a^		
	Yes, on my own	79 (78.2)	194 (56.7)		Ref	
	Yes, with the help of others	7 (6.9)	49 (14.3)		1.79 (0.50–6.34)	0.369
	No	15 (14.9)	99 (28.9)		1.88 (0.48–7.30)	0.360
6	Use of smartphone			0.008^a^		
	Yes, on my own	72 (71.3)	188 (54.9)		3.64 (1.13–11.72)	0.031^b^
	Yes, with the help of others	6 (5.9)	49 (14.3)		6.75 (1.47–31.07)	0.014^b^
	No	23 (22.8)	105 (30.7)		Ref	
7	Duration of phone use in a day (in hours)			0.004^a^		
	< 1	4 (3.9)	52 (15.2)		5.50 (1.08–27.89)	0.040^b^
	1−4	91 (90.1)	281 (82.2)		1.45 (0.47–4.44)	0.514
	> 4	6 (5.9)	9 (2.6)		Ref	
8	Availability of internet access at home			0.003^a^		
	Yes	28 (27.7)	151 (44.2)		1.42 (0.65–3.11)	0.382
	No	73 (72.3)	191 (55.9)		Ref	

^a^Chi-square *p*-value of < 0.05 that is included in the multivariate analysis.

^b^Significant factor (*p* < 0.05).

### Subgroup analysis based on prior telepharmacy use: users vs. non-users

Among patients who have used telepharmacy, being aged 51 to 60 years old (OR = 7.02; 95% CI = 2.04–24.12) or older than 60 years (OR = 6.80; 95% CI = 2.02–22.80), and ability to use smartphones with the help of others (OR = 23.62; 95% CI = 1.98–282.11) were significantly more likely associated with a lower willingness to use telepharmacy services. None of the characteristics showed a statistically significant association with low willingness to use telepharmacy among patients who have not used telepharmacy (Table 5).

**Table 5:** Subgroup analysis of factors associated with the low willingness to use telepharmacy.

No	Characteristics	Patients who have used telepharmacy (N = 196)		Patients who have not used telepharmacy (N = 247)	
		Odds ratio (95% CI)	*p*-value	Odds ratio (95% CI)	*p*-value
1	Age (in years)			N/A	N/A
	< 50	Ref			
	51–60	7.02 (2.04–24.12)	0.002^a^		
	> 60	6.80 (2.02–22.80)	0.002^a^		
2	Use of internet				
	Yes, on my own	1.57 (0.61–4.08)	0.351	1.24 (0.13–11.70)	0.851
	Yes, with the help of others	Ref		Ref	
	No	5.00 (1.01–24.72)	0.048	2.46 (0.21–28.95)	0.474
3	Use of cell phone				
	Yes, on my own	Ref		Ref	
	Yes, with the help of others	0.55 (0.10–3.18)	0.507	14.37 (0.89–230.88)	0.060
	No	2.37 (0.26–21.29)	0.441	2.46 (0.26–23.23)	0.430
4	Use of smartphone				
	Yes, on my own	5.07 (0.86–23.82)	0.072	8.80 (0.57–135.94)	0.119
	Yes, with the help of others	23.62 (1.98–282.11)	0.012^a^	1.43 (0.12–16.85)	0.773
	No	Ref		Ref	
5	Duration of phone use in a day (in hours)			N/A	N/A
	< 1	0.96 (0.11–8.66)	0.974		
	1–4	1.38 (0.37–5.14)	0.630		
	> 4	Ref			
6	Availability of internet access at home				
	Yes	1.00 (0.38–2.63)	0.996	2.88 (0.51–16.04)	0.228
	No	Ref		Ref	

^a^Significant factors (*p* < 0.05).

## Discussion

Our study revealed that almost all patients with chronic diseases showed low practices and willingness to use telepharmacy services, aligning with previous findings in Ethiopia^51^. This study revealed that several factors were significantly associated with the low willingness, including older age, inability to use the internet, ability to use smartphones, and low daily usage of smartphones.

The main reason for this observed low willingness and practice might be the limited digital literacy among patients^55^, defined as the capacity to effectively use digital technology and absorb information optimally^56^. Adequate digital literacy is a known facilitator for telemedicine adoption^57^, and conversely, difficulty adapting to technology significantly impacts interest in engaging with such services^58^. A key factor contributing to this resistance is chronic disease patients’ preference for maintaining their established routines of in-person appointments and direct treatment^59^, which may feel more comfortable than adapting to new methods of disease management, as these routines allow for direct physical assessment and comprehensive discussion crucial for managing complex and long-term conditions. This preference for conventional methods is particularly relevant in the context of the decline in telemedicine usage in Indonesia following the pandemic^60^, which may have led patients to perceive less urgency in utilizing these services, prompting a return to conventional methods of care, especially among those confident in their long-term disease management. People generally exhibit resistance to new innovative technologies due to their initial judgments about change, which significantly impacts decisions to use and often leads to the failure of IT-based health systems^61^. Furthermore, a significant barrier observed was patients’ high concern in terms of confidentiality when using telepharmacy services, that might threaten their personal information and privacy^62–64^. This concern directly impacts their trust in the technology, which is a foundational element for perceived usefulness and adoption within a Technology Acceptance Model (TAM) perspective^65^.

In contrast to our primary finding of low willingness, previous research demonstrated that individuals with chronic conditions can be more likely to continuously use telemedicine^66^. This was particulary evident during the COVID-19 pandemic, when patients at higher risk of infection were often required to use telemedicine for monitoring their health^67^, leading to increased exposure, positive attitudes, and improved perceptions towards its use^68^. This period may have temporarily elevated the relative advantage of telepharmacy due to immediate health necessity as stated by Diffusion Of Innovation (DOI) framework^69^. However, the post-pandemic decline in Indonesia seems to have reversed this trend, reinforcing the factors that favor conventional care in the current environment^60^,

In light of the fact that a majority of patients with chronic diseases in Indonesia, are elderly individuals^42,70^, understanding factors influencing their willingness to adopt telepharmacy is crucial. Our findings revealing that older patients are more likely to exhibit low willingness to access telepharmacy services align with numerous studies highlighting unfavorable perceptions of telepharmacy among older adults^62,71,72^. This association is likely driven by factors such as limited familiarity with technology^73,74^, potential physical and cognitive limitations impacting digital skill acquisition^75^, and a prevailing belief that their medical needs require in-person interactions^73,74^, particularly for chronic conditions requiring regular physical examination or complex medication reviews. Patients who struggle with digital skills will naturally find telepharmacy complex and difficult to navigate, thus reducing their intention to use it, which, from a TAM perspective, directly translates to a low perceived ease of use^76^. Addressing this requires tailored interventions, such as, tailored learning in a secure setting, clear instructions, and individualized instruction^77^, alongside a value-driven design considering their specific demands^78^.

A critical factor associated with a lower willingness to engage in telepharmacy identified in this study is the patient’s ability to use the internet and smartphones. Our study demonstrated that the inability to use these technologies, even with the assistance, was associated with low willingness to engage in telepharmacy, pointing to the fundamental barrier posed by digital literacy. Merely owning or using for basic functions like entertainment^79^ or communication purposes^80^ does not equate to the specific skills required for engagement of using telepharmacy services^81,82^. Telepharmacy platforms often involve specific interfaces for medication ordering, scheduling consultations, or securely sharing health information, which demand a higher level of digital proficiency than general internet use. This lack of specific digital health comptetence directly limits a patient’s capacity and confidence in accessing telepharmacy services, further reinforcing the concept of low perceived ease of use of TAM framework^83^, which is consistent with our finding that a large proportion of patients scored poorly in telepharmacy practices.

The association between minimum daily use of phones and low willingness to use telepharmacy services suggests that infrequent engagement with digital devices may reflect lower comfort, familiarity, or integration of technology into daily routines^80,84^. Unlike individuals who use smartphones extensively for various tasks, including health-related information^81^, patients with minimal daily use may lack the intuitive proficiency needed for telepharmacy applications. This limited digital exposure likely contributes to a lower willingness to adopt new digital health services such as telepharmacy that require more frequent interaction and comfort with the platform, especially when these services are integral to their ongoing chronic disease management. This highlighted a trialability challenge within the DOI framework for individuals less accustomed to digital interactions, as their limited opportunities to experiment with and gain confidence in new digital tools like telepharmacy hinder adoption^85^.

Low telepharmacy utilization among patients with chronic diseases has been documented in prior studies^26^. Beyond the individual factors identified, the type of chronic conditions may also significantly influence the acceptance of technology. Research indicates that a history of certain conditions like cancer, arthritis, or hypertension is linked to effective telemedicine use. In contrast, symptoms of depression or anxiety, along with heart disease, and diabetes, have been negatively associated with telemedicine engagement, potentially due to the technical challenges or the complexity of managing these conditions remotely^86,87^. Additionally, the presence of complicated medical conditions or comorbidities related to hypertension or diabetes can further hinder their participation in telemedicine^86,87^.

Our study found that chronic disease patients who had previously used telepharmacy were much more likely to have a high willingness to use it compared to patients who had not used it. However, it’s also worth noting that even among those who had used it, a majority still expressed low willingness. Our results showed that while factors like older age and assisted smartphone use were associated with willingness after using telepharmacy, these demographic or technology access characteristics did not appear to significantly predict the willingness to start using it among chronic disease patients who had not used telepharmacy in this study. This suggested that the barriers or motivators influencing the decision to start using telepharmacy might have been different from those influencing willingness after using it. This suggested that while usage experience increased willingness, there might still have been barriers or negative perceptions even after using it. This indicated that the direct experience with the telepharmacy service itself, or the specific health needs it addressed, was more influential on post-use willingness than general technology access or usage habits.

Telepharmacy offers significant benefits especially for patients with chronic diseases. Primarily, it serves as an approach to bridge existing gaps in healthcare delivery by enabling patients to receive comprehensive pharmaceutical services regardless of geographical location, thus reducing healthcare disparities and improving overall care^88^. Consequently, this enhanced accessibility facilitates greater access for patients to healthcare providers and resources for managing chronic diseases, which helps ensure continuity of care as patients transition from hospital to the community and provides constant support to family doctors^89^. Beyond improving access and continuity, telepharmacy services can also significantly enhance medication adherence among chronic disease patients. This is achieved by ensuring interventions facilitate communication between patients and health care teams through interactive reminders that provide individualized health monitoring and personalized medication information^90^. Furthermore, by tailoring services to patients’ individual health barriers and levels of health activation, telepharmacy can provide adherence support targeted at those with the greatest clinical need, potentially leading to further improved medication adherence^91^. Finally, telepharmacy services show promise for being cost-effective in the management of chronic diseases compared with standard care^92^, understanding that cost-effectiveness in health and medicine requires considering both financial costs and health outcomes, necessitating equivalent or improved clinical outcomes, not just reduced costs.

Assessing the current practices and willingness to use telepharmacy services is important for the long term in adopting and maintaining such services. Increasing utilization requires addressing underlying issues such as digital and health literacy^93^. Training programs highlighting the benefits of telepharmacy and providing clear instructions on its operation can improve patient acceptance, particularly among the elderly, and reduce unnecessary anxiety toward technology^94^. This training should include practical guidance on using telepharmacy tools for medication management, symptom reporting, and communicating with healthcare teams specifically for their chronic disease conditions. Improving individuals’ understanding of their diseases can also promote self-care and health-related behaviors^87^, potential openness to technology when a direct correlation between behaviors and outcomes is recognized^95^. Furthermore, supporting elderly patients’ digital competencies requires flexible program addressing their unique needs, such as the feeling of inferiority, lack of social assistance, anxiety, lack of prior experience, or physical restrictions^78^. Successful adoption of telepharmacy requires support from society^96^ and user-friendly design with clear instructions to provide accessibility and ease of use for patients^57^. To overcome the preference for conventional routines and facilitate in the socio-cultural context of Indonesia, where communal support is a strong tradition, integrating telepharmacy with conventional care practices could offer a balanced approach. This integration enables remote consultations and medication management, ensuring continuous care that is particularly crucial for managing chronic conditions requiring frequent adjustments or monitoring, potentially reducing the need for frequent hospital visits^97^. Telepharmacy can effectively complement existing support systems, including caregivers and family members, who can assist elderly patients in using telepharmacy services by setting up virtual consultations, managing medication schedules for multiple chronic conditions, and communicating with healthcare providers to ensure comprehensive care and enhance the overall care experience^90^. This integration is particularly beneficial for routine check-ups and medication refills, which can be efficiently managed through telepharmacy^89^. Importantly, telepharmacy should be framed not as a replacement for face-to-face interactions, but as a flexible and accessible option for patients who do not always require in-person visits^91^. For elderly patients, telepharmacy can help manage routine aspects of their care, while home care services and in-person consultations can address more complex needs. Different strategies might have been needed to promote initial adoption versus encouraging continuos use and high willingness; targeted interventions addressing awareness, perceived benefits, trust, and ease of access might have been more critical for encouraging initial uptake.

This study contributes to the understanding of the key factors of willingness to use telepharmacy among patients with chronic diseases in Indonesia and its current practice. The distinction allowed for the identification of specific factors affecting patients with chronic diseases, which could be used to develop a tailored and targeted intervention. Several limitations needed to be acknowledged. First, voluntary participation may have introduced selection bias, as patients with specific attitudes might have been more likely to participate in the study. Second, due to the cross-sectional nature of the analysis, causal inferences regarding the temporal associations between potential factors and outcomes could not be drawn. Third, since the data used were self-reported, desirability bias might have influenced responses, and a reporting bias existed related to the variable duration of phone use could lead to an overestimation of the association between phone usage duration and the willingness to use telepharmacy services. Fourth, the purposive selection of only seven CHCs and one hospital with the highest number of chronic diseases patients limits the direct generalizability to all chronic disease patients across Bandung City or other regions in Indonesia, as the experiences and willingness of patients in these facilities might differ from those in centers with lower patient loads. Fifth, we did not employ assisted questionnaires along with direct observation, especially for elderly participants, which may have limited the comprehensiveness of the data collected. This methodological decision could result in missing nuanced insights into the healthcare needs and challenges faced by the elderly population, potentially impacting the overall validity and applicability of the findings. Sixth, an absence of items specifically assessing disease-specific experiences within the questionnaire is noted. While the survey captured aspects of general telepharmacy practice and willingness among chronic disease patients, it did not explore the unique challenges, specific medication needs, or particular experiences associated with their individual chronic conditions that might uniquely influence patients’ perceptions, willingness, or actual practice regarding telepharmacy use. Seventh, some factors showed a wide range of 95% CIs indicating a higher level of uncertainty in the estimation of the population parameter. Eighth, the models used had a relatively low R-squared, implying that other unmeasured factors such as current expertise in technology^98^, anxiety related to technology^99^, type of telepharmacy services, and type of chronic diseases^100^, may be associated with low willingness to use telepharmacy services among patients with chronic diseases to varying levels. Finally, this study did not fully capture the subtle digital skills and confidence essential for effective telepharmacy engagement, making it challenging to definitively ascertain whether low willingness resulted from a lack of proficiency or other attitudinal factors. Future research would benefit from incorporating established digital health literacy assessment tools to provide a clearer understanding of patients’ baseline capabilities and to inform more targeted interventions.

## Conclusion

This study showed low practices and willingness to use telepharmcy services among patients with chronic diseases. Patients-specific factors such as older age, inability to use the internet, ability to use smartphones, and low daily usage of smartphones should be considered to facilitate the adoption of telepharmacy in Indonesia.

## Electronic supplementary material

Below is the link to the electronic supplementary material.

Supplementary Material 1

## Data Availability

The datasets used and/or analyzed are available from the corresponding authors upon reasonable request.
