# Supplementary material for: Willingness of patients with chronic diseases to use telepharmacy services in Bandung City, West Java, Indonesia
Source: Sci Rep. 2025 Jul 11;15:25078. doi: 10.1038/s41598-025-09688-3 (PMC12254323; doi:10.1038/s41598-025-09688-3)
Supplement: Supplementary file 1 — Supplementary Material 1 [file 41598_2025_9688_MOESM1_ESM.docx]

**SUPPLEMENTARY DATA**

**Table S1. STROBE Statement: Checklist of items that should be included in reports of cross-sectional studies**

|  | Item No | Recommendation | Page No |
| --- | --- | --- | --- |
| **Title and abstract** | 1 | (*a*) Indicate the study’s design with a commonly used term in the title or the abstract | 1-2 |
|  |  | (*b*) Provide in the abstract an informative and balanced summary of what was done and what was found | 2 |
| Introduction | | | |
| Background/rationale | 2 | Explain the scientific background and rationale for the investigation being reported | 3-4 |
| Objectives | 3 | State specific objectives, including any prespecified hypotheses | 4 |
| Methods | | | |
| Study design | 4 | Present key elements of study design early in the paper | 5 |
| Setting | 5 | Describe the setting, locations, and relevant dates, including periods of recruitment, exposure, follow-up, and data collection | 5 |
| Participants | 6 | (*a*) Give the eligibility criteria, and the sources and methods of selection of participants | 5 |
| Variables | 7 | Clearly define all outcomes, exposures, predictors, potential confounders, and effect modifiers. Give diagnostic criteria, if applicable | 5-8 |
| Data sources/ measurement | 8* | For each variable of interest, give sources of data and details of methods of assessment (measurement). Describe comparability of assessment methods if there is more than one group | 5-8 |
| Bias | 9 | Describe any efforts to address potential sources of bias | 7 |
| Study size | 10 | Explain how the study size was arrived at | 7 |
| Quantitative variables | 11 | Explain how quantitative variables were handled in the analyses. If applicable, describe which groupings were chosen and why | 6 |
| Statistical methods | 12 | (*a*) Describe all statistical methods, including those used to control for confounding | 8 |
|  |  | (*b*) Describe any methods used to examine subgroups and interactions | NA |
|  |  | (*c*) Explain how missing data were addressed | NA |
|  |  | (*d*) If applicable, describe analytical methods taking account of sampling strategy | NA |
|  |  | (*e*) Describe any sensitivity analyses | NA |
| Results | | | |
| Participants | 13* | (a) Report numbers of individuals at each stage of study—eg numbers potentially eligible, examined for eligibility, confirmed eligible, included in the study, completing follow-up, and analysed | 9 |
|  |  | (b) Give reasons for non-participation at each stage | NA |
|  |  | (c) Consider use of a flow diagram | NA |
| Descriptive data | 14* | (a) Give characteristics of study participants (eg demographic, clinical, social) and information on exposures and potential confounders | 10-11 |
|  |  | (b) Indicate number of participants with missing data for each variable of interest | NA |
| Outcome data | 15* | Report numbers of outcome events or summary measures | 9-11 |
| Main results | 16 | (*a*) Give unadjusted estimates and, if applicable, confounder-adjusted estimates and their precision (eg, 95% confidence interval). Make clear which confounders were adjusted for and why they were included | 9-11 |
|  |  | (*b*) Report category boundaries when continuous variables were categorized | 9-10 |
|  |  | (*c*) If relevant, consider translating estimates of relative risk into absolute risk for a meaningful time period | NA |
| Other analyses | 17 | Report other analyses done—eg analyses of subgroups and interactions, and sensitivity analyses | NA |
| Discussion | | | |
| Key results | 18 | Summarise key results with reference to study objectives | 12-17 |
| Limitations | 19 | Discuss limitations of the study, taking into account sources of potential bias or imprecision. Discuss both direction and magnitude of any potential bias | 17 |
| Interpretation | 20 | Give a cautious overall interpretation of results considering objectives, limitations, multiplicity of analyses, results from similar studies, and other relevant evidence | 12-17 |
| Generalisability | 21 | Discuss the generalisability (external validity) of the study results | 12-15 |
| Other information | | | |
| Funding | 22 | Give the source of funding and the role of the funders for the present study and, if applicable, for the original study on which the present article is based | 18 |

*Give information separately for exposed and unexposed groups.

**Tabel S2. Information about patient’s chronic diseases (N = 443)**

| **No** | **Chronic disease** | **N (%)** | **Multiple chronic disease (dyad)** | **N (%)** | **Multiple chronic disease (triad)** | **N (%)** |
| --- | --- | --- | --- | --- | --- | --- |
| 1 | Bipolar | 1 (0.2) | Diabetes and cardiovascular disease | 3 (0.7) | Diabetes, hypertension, cardiovascular, and ocular disease | 1 (0.2) |
| 2 | Diabetes | 76 (17.2) | Diabetes and hypercholesterolemia | 12 (2.7) | Hypertension, diabetes, and dyslipidemia | 5 (1.1) |
| 3 | Dyslipidemia | 1 (0.2) | Dyslipidemia and vertigo | 1 (0.2) | Hypertension, diabetes, asthma, cardiovascular, and neurological disease | 1 (0.2) |
| 4 | Kidney failure | 1 (0.2) | Hypertension and gout disease | 1 (0.2) | Hypertension, diabetes, and cardiovascular disease | 5 (1.1) |
| 5 | Hepatitis B | 1 (0.2) | Hypertension and asthma | 2 (0.5) | Hypertension, diabetes, hypercholesterolemia, and cardiovascular diseases | 1 (0.2) |
| 6 | Hypertension | 158 (35.7) | Hypertension and hypotension | 1 (0.2) | Hypertension, diabetes, hypercholesterolemia, and gout diseases | 1 (0.2) |
| 7 | Hyperthyroid | 1 (0.2) | Hypertension and diabetes | 54 (12.2) | Hypertension, diabetes, and asthma | 3 (0.7) |
| 8 | Cardiovascular disease | 10 (2.3) | Hypertension and dyslipidemia | 5 (1.1) | Hypertension, diabetes, and hypercholesterolemia | 16 (3.6) |
| 9 | Hypercholesterolemia | 2 (0.5) | Hypertension and cardiovascular disease | 24 (5.4) | Hypertension, diabetes, and gastrointestinal disease | 3 (0.7) |
| 10 | COPD | 3 (0.7) | Hypertension and hypercholesterolemia | 25 (5.6) | Hypertension, diabetes, and COPD | 1 (0.2) |
| 11 | Stroke | 1 (0.2) | Hypertension and stroke | 1 (0.2) | Hypertension, diabetes, and prostate disease | 1 (0.2) |
| 12 | Neurological disease | 2 (0.5) | Hypertension and neurological disease | 3 (0.7) | Hypertension, diabetes, and kidney failure | 1 (0.2) |
| 13 | Tuberculosis | 1 (0.2) | Cardiovascular disease and kidney failure | 1 (0.2) | Hypertension, diabetes, and lung disease | 1 (0.2) |
| 14 |  |  | Cardiovascular and gastrointestinal disease | 1 (0.2) | Hypertension, cardiovascular, and neurological disease | 1 (0.2) |
| 15 |  |  | Cardiovascular and neurological disease | 1 (0.2) | Hypertension, hypercholesterolemia, asthma, and cardiovascular disease | 1 (0.2) |
| 16 |  |  | Hypercholesterolemia and cardiovascular disease | 1 (0.2) | Hypertension, hypercholesterolemia, gout, and cardiovascular disease | 1 (0.2) |
| 17 |  |  | COPD and cardiovascular disease | 1 (0.23) | Hypertension, hypercholesterolemia, knee, and lower back disease | 1 (0.2) |
| 18 |  |  |  |  | Hypertension, hypercholesterolemia, and asthma | 1 (0.2) |
| 19 |  |  |  |  | Hypertension, hypercholesterolemia, and cardiovascular disease | 1 (0.2) |
| 20 |  |  |  |  | Hypertension, hypercholesterolemia, and stroke | 2 (0.5) |
| **Subgroup Total** | | 258 (58%) | 137 (31%) | | 48 (11%) | |
|  |  |  | 185 (42%) | | | |
| **Total** | | **443 (100%)** | | | | |

**Table S3. Questionnaire of the current practice of telepharmacy use**

| **No** | **Current Practice Statements/Questions** | **Category** |
| --- | --- | --- |
| 1. | Have you ever searched online for information about a disease or medical problem that you have? | - Yes - No |
| 2. | Have you ever searched online for information about the doctor you are having appointments with? | - Yes - No |
| 3. | Have you ever typed information on an application about your dietary, physical exercise, and overall lifestyle modifications? | - Yes - No |
| 4. | Have you ever typed information on an application about a chronic illness you have? | - Yes - No |
| 5. | Have you ever renewed a prescription online? | - Yes - No |
| 6. | Have you ever consulted your doctor/pharmacist online? | - Yes - No |
| 7. | Have you ever used a personal online health record for your health? | - Yes - No |
| 8. | Have you ever looked at a medical test result online? | - Yes - No |
| 9. | Have you ever used a device that measures health information (like blood pressure; and blood glucose level) that connects to your mobile/website application? | - Yes - No |
| 10. | Have you ever posted anything online about your health condition or health care? | - Yes - No |
| 11. | Have you ever joined an online group that is for a health issue that you have? | - Yes - No |
| 12. | Have you ever booked an appointment with doctors/pharmacists online? | - Yes - No |

**Table S4. Questionnaire of the willingness to use telepharmacy**

| **No** | **Willingness Statements/Questions** | **Category** |
| --- | --- | --- |
| 1. | Confident about your own health management. | - Very confident - Somewhat confident - Not too confident - Not at all confident |
| 2. | Are you familiar with telepharmacy? | - Yes - Yes, I have heard, but I do not know the details - No |
| 3. | Interest tracking information about chronic illness | - Yes - No |
| 4. | Interest track information to track your diet and calories | - Yes - No |
| 5. | Interest information track your exercise | - Yes - No |
| 6. | Interest information track reminds you when to take prescriptions | - Yes - No |
| 7. | Interest information track reminds you when you need tests | - Yes - No |
| 8. | Would you be interested in using this type of telepharmacy if it were from your doctor? | - Yes - No |
| 9. | Would you be interested in using this type of telepharmacy if it were from the hospital you use? | - Yes - No |
| 10. | Would you be interested in using this type of telepharmacy if it were from a pharmacist? | - Yes - No |
| 11. | Would you be interested in using this type of telepharmacy if it were from your health insurance plan? | - Yes - No |
| 12. | Would you be interested in using this type of telepharmacy if it were from a government group? | - Yes - No |
| 13. | Would you be interested in using this type of telepharmacy if it were from a nurse? | - Yes - No |
| 14. | Would you be interested in using this type of telepharmacy if it were from a company like Google or Apple? | - Yes - No |
| 15. | In general, if your health information were online, how worried would you be about the privacy and confidentiality of your information? | - Very worried - Somewhat worried - Not too worried - Not at all worried |
| 16. | Currently, how much you are interested in using telepharmacy (application, website)? | - Very interested - Interested - A little bit interested - Not at all interested |
